# Effects of Ovariectomy and Sex Steroids on Prolactin Synthesis

**DOI:** 10.1155/ije/8883536

**Published:** 2025-12-11

**Authors:** Zhuoma Cairang, Haruhiko Kanasaki, Tuvshintugs Tumurbaatar, Susdiaman S. Yacca, Aki Oride, Hiroe Okada, Satoru Kyo

**Affiliations:** ^1^ Department of Obstetrics and Gynecology, Shimane University Faculty of Medicine, Izumo, 693-8501, Japan, shimane-u.ac.jp

**Keywords:** estradiol, HPG axis, ovariectomy, prolactin, TRH

## Abstract

Prolactin (PRL) plays a variety of roles to maintain female reproductive functions. In female rats, *Prl* mRNA expression in the anterior pituitary was significantly suppressed by OVX. The OVX‐induced inhibition of PRL expression was recovered by estradiol (E2) supplementation after OVX. Supplementation of progesterone (P4) and the androgen dihydrotestosterone (DHT) did not prevent the OVX‐induced decrease of PRL expression. When E2 was administered to ovary‐intact rats, *Prl* expression was significantly increased, but neither P4 nor DHT modulated its expression. In experiments using the PRL‐producing GH3 cell line, a higher concentration of E2 significantly increased *Prl* expression; however, neither P4 nor DHT modulated its expression in these cells. Gene expression of thyrotropin‐releasing hormone (TRH), a PRL‐releasing factor within the hypothalamus, was unchanged by OVX. Similarly, OVX had no effect on the hypothalamic expression of the gene encoding tyrosine hydroxylase, a marker of dopamine that inhibits PRL. In addition, mRNA expression of TRH receptor and dopamine D2 receptor did not change by OVX within the pituitary. Moreover, the expression of follistatin (*Fst*) mRNA was increased following OVX and decreased with E2 supplementation in the anterior pituitary. However, FST had no effect on TRH‐induced PRL synthesis. Our current observations suggest that the OVX‐induced reduction of PRL principally depends on the depletion of E2. E2 has a direct effect on PRL‐producing lactotrophs and increases PRL levels without affecting hypothalamic PRL‐releasing/inhibiting factors.

## 1. Introduction

Reproductive function is primarily controlled by pituitary gonadotropins, luteinizing hormone (LH), and follicle‐stimulating hormone (FSH). In addition, prolactin (PRL), which is secreted from pituitary lactotrophs, possesses numerous functions related to reproduction [1, 2]. Generally, PRL secretion is thought to be primarily regulated by dopaminergic inhibition originating from the hypothalamus [3]. In addition, thyrotropin‐releasing hormone (TRH), which is released from the hypothalamus, is considered as a PRL‐releasing factor that stimulates PRL production. However, its role as a physiological regulator remains unclear [4]. In addition, other hypothalamic peptides, including vasoactive intestinal polypeptide [5], serotonin [6], oxytocin [7], and pituitary adenylate cyclase‐activating polypeptide [8], participate in the control of PRL levels.

Evidence from earlier studies has demonstrated that sex steroids are also involved in the control of PRL. In some women, PRL levels are elevated during the follicular phase compared to those in the luteal phase and lower during the luteal phase than during the follicular phase [9]. Circulating PRL levels gradually increase throughout pregnancy [10], which has generally been attributed to the stimulatory effect of the hormonal milieu of pregnancy, primarily by the effect of estradiol (E2) on pituitary PRL‐producing lactotrophs. Furthermore, E2 has a negative influence on dopamine neurons and increases PRL secretion [11]. E2 has also been shown to decrease the number of dopamine receptors [12], while it increases the number of TRH receptors in anterior pituitary cells [13].

Circulating E2 level of reduction increases the production of the pituitary gonadotropins LH and FSH and stimulates the ovary to produce more E2 by negative feedback mechanisms in the hypothalamic–pituitary–gonadal (HPG) axis. At the present, it is widely recognized that hypothalamic kisspeptin neurons detect decreases in the E2 level and stimulate pulsatile secretion of gonadotropin‐releasing hormone (GnRH) [14]. In addition, ovarian sex steroid E2, progesterone (P4), and androgens also contribute in the regulation of the HPG axis [15]. Hyperprolactinemia can lead to menstrual disturbance, infertility, and galactorrhea [16]. Moreover, PRL and its receptor are expressed within the ovaries, and it has been hypothesized that excess PRL negatively impacts steroid secretion [17]. Thus, it is plausible that PRL and the HPG axis are strongly involved with each other.

Ovariectomy (OVX)‐induced depletion of sex steroids stimulates the secretion and synthesis of pituitary LH and FSH through a negative feedback mechanism that is mediated by kisspeptin and GnRH neurons in the hypothalamus. A previous study reported that OVX reduces the volume of pituitary mammotrophs in rats and that E2 supplementation after OVX exerted stimulatory changes in mammotrophs [18]. However, the effects of OVX on pituitary PRL and its mechanisms remain incompletely understood. In the present study, we examined the effects of OVX on the secretion and synthesis of PRL. We also evaluated the effects of sex steroid supplementation on PRL expression after OVX. Furthermore, we examined the direct effects of the sex steroids E2 and P4 and the androgen dihydrotestosterone (DHT) on PRL gene expression, using PRL‐producing cell models. In addition, changes in hypothalamic factors that are involved in PRL synthesis as well as their receptor expression within the pituitary gland were also investigated.

## 2. Materials and Methods

### 2.1. Materials

Chemicals and reagents used in this study were obtained from the listed sources: high‐glucose Dulbecco’s modified Eagle’s medium (DMEM, 4.5 g/L glucose), penicillin–streptomycin, fetal bovine serum (Invitrogen, Carlsbad, CA), water‐soluble E2, P4, 5α‐DHT, TRH, and follistatin (FST) (Sigma‐Aldrich Co., St. Louis, MO).

### 2.2. In Vivo Experiments

Six‐week‐old Wistar female rats (average body weight, 150 ± 5 g) were housed under a 12‐h light/dark cycle at 20°C–25°C, with free access to feed (CE‐2; CLEA Japan, Tokyo, Japan) and water. The rats were housed two per cage, and vaginal smears were examined daily at 10 a.m. to monitor their estrous cycle. After 1 week of observation, rats were anesthetized via intraperitoneal injection of a combination of butorphanol (2.5 mg/kg), midazolam (2 mg/kg), and medetomidine (0.15 mg/kg), followed by bilateral OVX. Rats that underwent sham surgery (ovary‐sparing) served as the control group. After OVX, the rats were allowed to recover for 1 week before receiving subcutaneous pellet or injection for 7 days containing either 0.25 mg E2 or 50 mg P4 (Innovative Research of America, Sarasota, FL) or daily subcutaneous injection of 25 mg/kg of DHT in 140 μL of sesame oil. All doses and the duration of treatment were decided based on previous studies [19–22]. In the experiments using rats with intact ovaries, sex steroids were administered for 7 days without performing OVX, and control rats received daily injections of 140 μL of sesame oil. The rats were euthanized under isoflurane anesthesia (Fujifilm Wako Pure Chemical Corp., Osaka, Japan), and the entire brain and anterior pituitary gland were removed and divided evenly along the sagittal section. One‐half of the brain and the anterior pituitary gland were preserved for future experiments, while the remaining half was used for this study. The hypothalamus was dissected while referring to a rat brain atlas [23]. The hypothalamus was divided coronally into anterior and posterior parts for further experimental analysis. This protocol has been approved by the Ethics Review Committee of the Shimane University Experimental Animal Center for Integrated Research (IZ6‐41).

### 2.3. Cell Culture

GH3 cells (CCL‐82.1, American Type Culture Collection; cell passage numbers 25–35) were plated in 35‐mm culture dishes at a density of 2 × 10^5^ cells/dish. Cell cultures were maintained in high‐glucose DMEM (4.5 g/L glucose), supplemented with 1% of penicillin and streptomycin and 10% thermal inactivated fetal bovine serum, and incubated at 37°C in a humidified atmosphere of 5% CO_2_. After 48 h, the cell culture medium was replaced with high‐glucose DMEM, and either the control group (no additives) or the test compounds E2, P4, DHT, or TRH at concentrations of 10 nM, 100 nM, or 1 μM, or FST at a concentration of 10 μg/mL were added for 24 h.

### 2.4. RNA Extraction, Reverse Transcription, PCR, and Quantitative Real‐Time (RT) PCR

Total RNA was extracted from 10 to 20 mg of rat hypothalamic tissue or from GH3 cells at 70%–80% confluency, using TRIzol LS (Thermo Fisher Scientific, Waltham, MA). RNA purity was confirmed by A260/A280 ratios greater than 1.8 measured using a NanoDrop spectrophotometer (Thermo Fisher Scientific). Complementary DNA synthesized from 1.0 μg of total RNA was reverse‐transcribed using an oligo‐dT primer (Promega, Madison, WI) and prepared using a First‐Strand cDNA Synthesis Kit (Invitrogen) and reverse transcription buffer. The reaction mixture final volume is 10 μL, containing a 200 U RNase inhibitor/human placenta ribonuclease inhibitor, 10 mM dithiothreitol, and 1 mM each dNTP (#2310; Takara, Tokyo, Japan). The reaction was heated at 37°C for 60 min. Quantification of *Prl*, *Trh*, tyrosine hydroxylase (*Th*), TRH receptor (*Trhr*), and dopamine D2 receptor (*D2r*) mRNA quantification was performed by quantitative RT‐PCR (Takara TP900; Takara‐Bio, Tokyo, Japan) using FastStart Master Mix and Universal ProbeLibrary Probes (Roche Diagnostics, Mannheim, Germany). The PCR primers were designed based on the published nucleotide sequences [24, 25], and the list of primers used in this experiment is provided in Table 1. The simultaneous measurement of target mRNAs and *Gapdh* enabled normalization at the transcript levels. Each set of primers included a no‐template control. Each reaction included 13.5 μL of total volume with 2 μL volume of a 50 ng/μL concentration cDNA. The thermal cycle conditions were as follows: After denaturing at 94°C for 10 min, we repeat 40 cycles of 15 s at 94°C followed by 1 min at 55°C. Following the reaction, we perform melting curve analysis (55°C–95°C). To determine PCR efficiency, cDNA was serially diluted 10‐fold according to previously reported methods [26]. PCR conditions were optimized to achieve an efficiency of 95% or higher, and only reactions with efficiencies ranging from 95% to 105% were incorporated into subsequent analyses. The relative differences in cDNA concentration between the baseline state and the experimental state were calculated using the comparative threshold cycle (Ct) method [27]. In summary, for each sample, ΔCt was calculated using the following equation for normalization against the internal control: ΔCt = Ct (gene) − Ct (Gapdh). To obtain the difference between the experimental and control conditions, ΔΔCt was calculated as ΔCt (sample) − ΔCt (control). Relative mRNA levels were calculated using the following equation: fold change = 2^ΔΔCt^.

**Table 1:** Primer sequence used in this experiment.

Gene name	Primer sequence (5′–3′)	Amplicon size (bp)
*Prl*	Forward, AAT GAC GGA AAT AGA TGA TTG	538
	Reverse, CCA GTT ATT AGT TGA AAC AGA	

*Trh*	Forward, AAA CCC CTC CCT TAC CCA CT	150
	Reverse, GAA GAA CCG TCT TGG CCA GT	

*Th*	Forward, TCC CCT GGT TCC CAA GAA AAG	546
	Reverse, CAA ATG TGC GGT CAG CCA AC	

*Trhr*	Forward, AGA TGC TCG CAG TGG TTG TA	258
	Reverse, GGG CCA CAC TGT AGT TAG CA	

*D2r*	Forward, TGG GAG TTT CCC AGT GAA CAG	458
	Reverse, ATG TGA AGG CGC TGT AGA GG	

*Gapdh*	Forward, ACC ACA GTC CAT GCC ATC AC	452
	Reverse, TCC ACC ACC CTG TTG CTG TA	

### 2.5. Western Blot Analysis

Tissue samples, which were previously frozen right after brain extraction of anterior pituitary (unmeasurable) or posterior hypothalamus (10–20 mg), were placed on ice and homogenized in radioimmunoprecipitation assay (RIPA) buffer (1% NP‐40, 0.1% sodium dodecyl sulfate and 0.5% sodium deoxycholate, in phosphate‐buffered saline) with 30 mg/mL of aprotinin, 0.1 mg/mL of phenylmethyl sulfonyl fluoride, and 1 mM sodium orthovanadate. After homogenizing for 10 s, the lysates were centrifuged at 14,000 × g at 4°C for 10 min. Cell lysate protein concentrations were measured using the Bradford method. Denatured protein in Laemmli buffer, boiled at 95°C for 5 min (15 μg per well), was resolved by 12% sodium dodecyl sulfate‐polyacrylamide gel electrophoresis, separated by electrophoresis for 35 min at 200 V, and transferred to polyvinylidene difluoride membranes (Hybond‐P PVDF; Amersham Biosciences, Little Chalfont, UK), using semidry transfer at 15 V for 30 min. The membranes were blocked for 2 h at room temperature in Blotto (5% milk in Tris‐buffered saline). Then, the membranes were incubated with previously published mouse monoclonal anti‐PRL antibody (1:100 dilution; sc271773, Santa Cruz Biotechnology, Dallas, TX) [28] at 4°C overnight and washed three times with 1% Tween/Tris‐buffered saline for 10 min. Subsequently, the membrane was incubated with antimouse horseradish peroxidase‐labeled secondary antibody (1:10,000 dilution) at room temperature for 1 h in the blot solution, and additional cleaning was performed as necessary. After enhanced chemiluminescence detection (Amersham Biosciences), the membrane was exposed to X‐ray film (Fujifilm, Tokyo, Japan). Protein expression levels were quantified by densitometry (ImageJ; National Institutes of Health, Bethesda, MD), and the protein band intensities were normalized to the intensity of anti‐β actin mouse monoclonal antibody (1 µg/mL dilution; ab8226, Abcam, Japan) [29] to correct for protein loading. β‐actin was detected on the same membrane after stripping with a stripping buffer (sc‐281698; Santa Cruz Biotechnology).

### 2.6. Hormone Measurement

Blood was collected via pericardial puncture during slaughter (10:00 a.m. to 12:00 p.m.) and clotted at room temperature for 1 hour and then centrifuged at 3000 × g for 15 min to separate the serum. The separated serum was stored at −20°C. The serum PRL concentration was determined using a Rat PRL ELISA Kit (catalog number: ERA50RB; Thermo Fisher Scientific) following the manufacturer’s protocol. The kit has a sensitivity of 0.41 ng/mL with an intraassay CV of < 10% and an interassay CV of < 12%. Absorbance was measured at 450 nm using a spectrophotometer, and sample concentrations were calculated based on a standard curve ranging from 0.41 to 100 ng/mL. Values are reported in ng/mL in serum.

### 2.7. Luciferase Assay

The plasmid reporter constructs were generated by fusing the −609/+12 region of the *Prl* gene to the firefly luciferase cDNA within the pGL3 vector (*Prl*‐Luc) [30]. A total of 2 × 10^5^ GH3 cells were transiently transfected by electroporation [31] with 2.0 μg/well of reporter construct and 0.1 μg/well pRL‐TK (Promega), using a Gene Pulser Xcell (Bio‐Rad Laboratories, Herculesm, CA) at 240 V with a 20 ms pulse duration, using a 2 mm cuvette with 500 μL of PBS–glucose, and plated in 35‐mm cell culture dishes. After 48 h, the culture medium was replaced with serum‐free high‐glucose DMEM and stimulated with or without (control) stimulation reagents of E2, P4, DHT, and TRH at 10 nM, 100 nM, and 1 μM or FST at 10 μg/mL for 6 h; then, cells were washed with ice‐cold phosphate‐buffered saline and lysed with passive lysis buffer (Promega). Cell debris was precipitated by centrifugation at 14,000 × g for 10 min at 4°C. *Firefly* and *Renilla* luciferase activities in the supernatants were measured using the Dual‐Luciferase Reporter Assay System using a luminometer (TD‐20/20; Promega). *Firefly* luciferase activity was normalized against *Renilla* luciferase activity to correct for transfection efficiency, and the results are presented as a fold increase relative to the unstimulated control. All experiments were performed independently in triplicate, with each experiment repeated three times.

### 2.8. Statistical Analysis

All experiments were performed independently at least three times. Each experiment within each experimental group was conducted using duplicate samples (quantitative RT‐PCR, western blotting). When confirming mRNA or protein expression, two samples were measured in duplicate. The mean ± standard error of the mean (SEM) was calculated from four sets of data. Using the three mean values obtained from three independent experiments, the data were statistically analyzed, and the mean SEM was calculated. In the luciferase assay or ELISA assay, each experiment was repeated three times, and each sample was measured three times. The mean ± SEM was calculated from the three means obtained from the three independent experiments. Statistical analysis was performed using one‐way analysis of variance (ANOVA) followed by Duncan’s multiple comparison test. *p* < 0.05 was considered statistically significant. All analyses were performed using Prism ver. 6.07 (GraphPad Software, San Diego, CA).

## 3. Results

### 3.1. Effect of OVX on Pituitary Prl mRNA Expression

**Figure 1:**
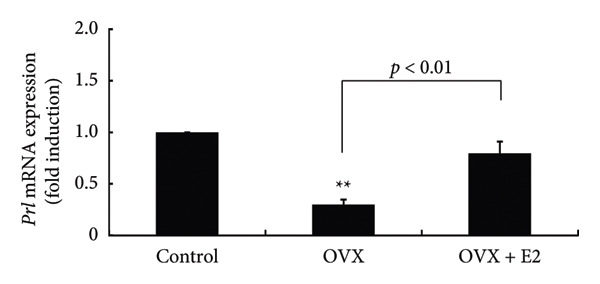
Effects of OVX and E2 replacement on *Prl* expression. OVX was performed on female rats at 6 weeks of age. Alternatively, a 0.25 mg E2 pellet was implanted subcutaneously after OVX (OVX + E2). As a control, sham‐operated (ovarian preservation) rats were served. Seven days later, the rats were euthanized, and the anterior pituitary gland dissected. Total mRNA was extracted from the pituitary tissue and subsequently reverse‐transcribed. *Prl* mRNA levels were measured by quantitative RT‐PCR. Samples from each experimental group were analyzed in duplicate and normalized against the mRNA level of endogenous *Gapdh* gene. The results are expressed as fold induction over control and presented as the mean ± SEM. Statistical significance was determined at ^∗∗^ *p* < 0.01 compared to control. Significant (*p* < 0.01) difference was observed between OVX and OVX + E2 rats in *Prl* expression.

To determine the effect of OVX on pituitary *Prl* expression, female rats were ovariectomized and pituitary *Prl* mRNA levels were determined. Following OVX, basal *Prl* mRNA expression within the anterior pituitary gland was significantly reduced to 0.30 ± 0.05‐fold (*p* < 0.01). Supplementation with E2 after OVX significantly recovered the OVX‐induced decrease in *Prl* expression (*p* < 0.01) (Figure 1).

### 3.2. Effect of Sex Steroid Supplementation After OVX on Pituitary PRL Expression

We examined pituitary PRL expression after OVX and the effects of E2, P4, and DHT supplementation. Western blot analysis showed that pituitary PRL expression was significantly decreased by OVX (*p* < 0.01). E2 supplementation after OVX significantly prevented the decrease in PRL expression compared with the control (sham‐operated) (*p* < 0.01); however, neither P4 nor DHT prevented the decrease in PRL levels after OVX (Figures 2(a) and 2(b)). Similarly, PRL serum levels were significantly decreased in OVX rats (*p* < 0.01), and supplementation with E2, but not P4 or DHT, after OVX prevented the decrease in serum PRL (*p* < 0.01) (Figure 2(c)).

Figure 2Effects of OVX and replacement of E2, P4, and DHT on PRL expression following OVX. OVX was performed on 6‐week‐old female rats. Following OVX, a 0.25 mg E2 pellet (OVX + E2) or 50 mg P4 pellet (OVX + P4) was implanted subcutaneously. Alternatively, 25 mg/kg of DHT was administered daily by subcutaneous injection after OVX (OVX + DHT). As a control, sham‐operated (ovarian preservation) rats served. Seven days later, the rats were euthanized and the anterior pituitary glands were dissected. (a) The anterior pituitary tissue lysate (15 μg protein) was analyzed by sodium dodecyl sulfate‐polyacrylamide gel electrophoresis, followed by immunoblotting and reaction with anti‐PRL antibody. β‐actin was detected as an internal control. Rat brain tissue‐derived protein was used as a positive control. (b) Band intensity was quantified by densitometric analysis using ImageJ software, and differences in PRL protein levels were determined by normalization to β‐actin levels. (c) Serum PRL levels were measured using a rat PRL enzyme‐linked immunosorbent assay. The results are expressed as the fold change in expression relative to nonstimulated cells. Data are presented as the mean ± SEM from three independent experiments conducted using duplicate samples. Statistical significance was determined at ^∗∗^ *p* < 0.01 and ^∗^ *p* < 0.05 compared to control. Significant difference was observed between OVX and OVX + E2 rats on *Prl* expression (b, c).

**Figure:**
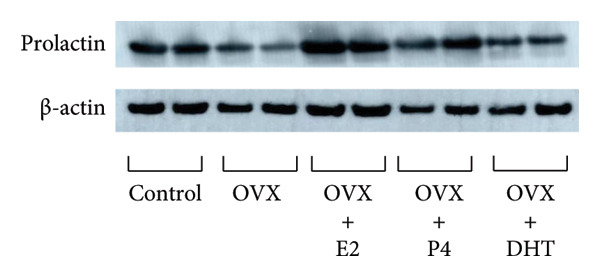
(a)

**Figure:**
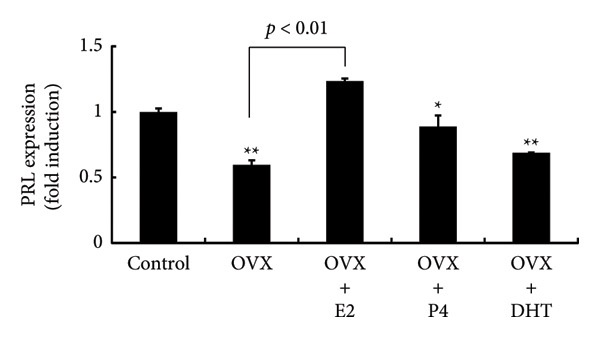
(b)

**Figure:**
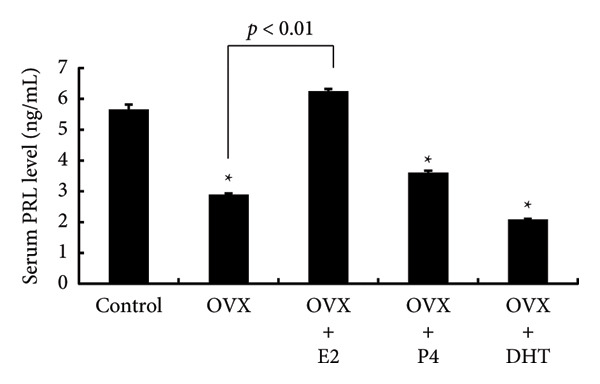
(c)

### 3.3. Effects of Sex Steroids in Ovary‐Intact Female Rats

Next, we examined the effects of sex steroid administration on PRL expression in ovary‐intact female rats. Pituitary PRL expression was increased by E2 administration by 1.69 ± 0.052‐fold in ovary‐intact rats (*p* < 0.05). However, neither P4 nor DHT administration modulated PRL expression (Figures 3(a) and 3(b)). We confirmed that the estrous cycle completely disappeared by administration of P4 and DHT (data not shown).

Figure 3Effects of E2, P4, and DHT administration on PRL expression in rats with retained ovaries. Seven‐week‐old Wistar female rats with intact ovaries received either pellets containing 0.25 mg of E2 or 50 mg of P4, or daily subcutaneous injections of 25 mg/kg of DHT. The control group rats were injected with 140 μL of sesame oil daily. (a) The anterior pituitary tissue lysate (15 μg protein) was analyzed by sodium dodecyl sulfate‐polyacrylamide gel electrophoresis, followed by immunoblotting and reaction with anti‐PRL antibody. β‐actin was detected as the internal control. Rat brain tissue‐derived protein was used as a positive control. The bands were visualized using a horseradish peroxidase‐conjugated secondary antibody. (b) Band scanning densitometry was performed using ImageJ, and differences in PRL protein levels were determined by normalization to β‐actin levels. Data are presented as the mean ± SEM from three independent experiments conducted using duplicate samples. Statistical significance was determined at ^∗^ *p* < 0.05 compared to control.

**Figure:**
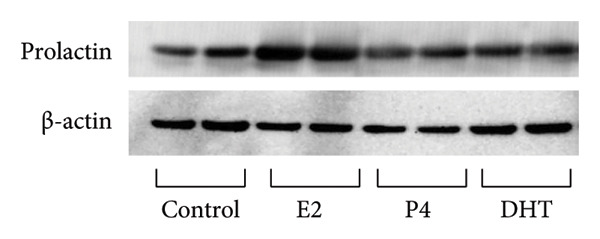
(a)

**Figure:**
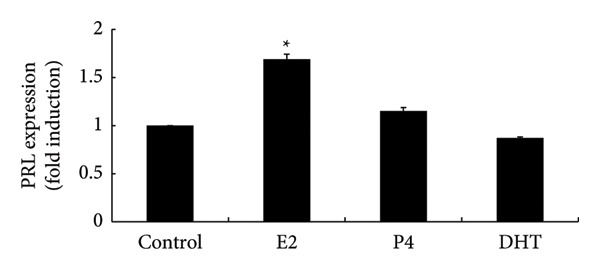
(b)

### 3.4. Effects of Sex Steroids on PRL Expression in PRL‐Producing GH3 Cells

To investigate the direct effects of sex steroids, we stimulated rat somatolactotroph GH3 cells with sex steroids. *Prl* promoter activity, which was determined by luciferase assays, was not modulated by E2, P4, or DHT, even when higher concentrations were applied (Figures 4(a), 4(b), and 4(c)). However, *Prl* mRNA expression significantly increased with E2 stimulation in GH3 cells by 3.73 ± 0.38‐fold compared to nonstimulated control (*p* < 0.05) (Figure 4(d)). Neither P4 nor DHT stimulated *Prl* mRNA expression in these cells (Figures 4(e) and 4(f)).

Figure 4Effects of E2, P4, and DHT on *Prl* promoter activity and mRNA expression in GH3 cells. (a, b, c) GH3 cells were transfected with 2.0 μg *Prl*‐Luc and 0.1 μg pRL‐TK. After 48 h of transfection, the cells were treated with the indicated concentrations of E2 (a), P4 (b), and DHT (c) for 6 h. A luciferase assay was performed to evaluate *Prl* promoter activity. This activity was normalized by *Renilla* luciferase activity and expressed as a fold increase in activation relative to the unstimulated control group. (d, e, f) GH3 cells were stimulated with the indicated concentrations of E2 (d), P4 (e), and DHT (f) for 24 h. Following stimulation, total mRNA was extracted and reverse‐transcribed into cDNA. Quantitative real‐time PCR was performed to assess *Prl* mRNA expression. Samples from each experimental group were processed in duplicate and normalized against mRNA levels of the housekeeping gene *Gapdh*. The results are presented as the induction ratio relative to nonstimulated cells. Data are shown as mean ± SEM (based on three independent experiments, each performed in triplicate). Statistical significance was determined at ^∗^ *p* < 0.05 compared to control.

**Figure:**
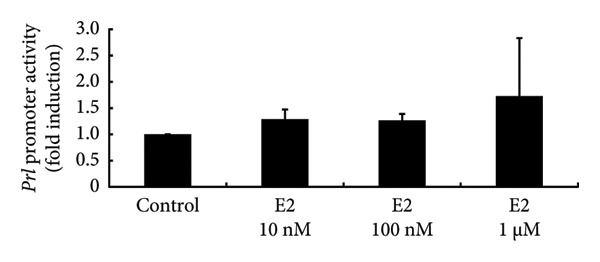
(a)

**Figure:**
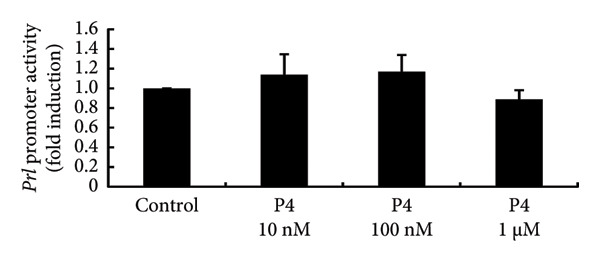
(b)

**Figure:**
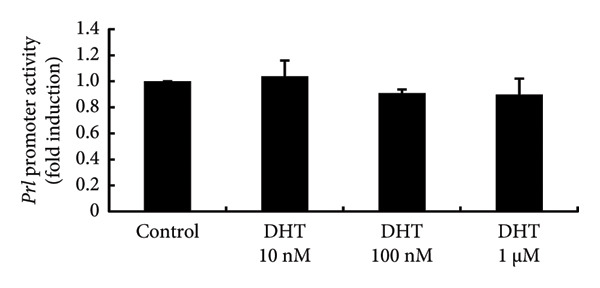
(c)

**Figure:**
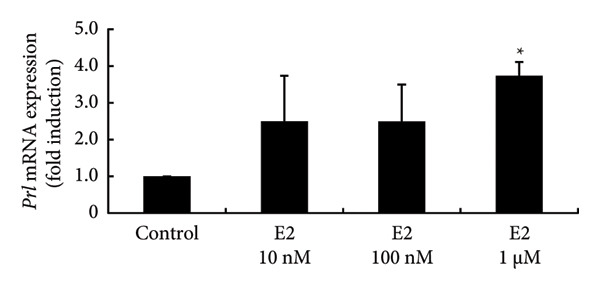
(d)

**Figure:**
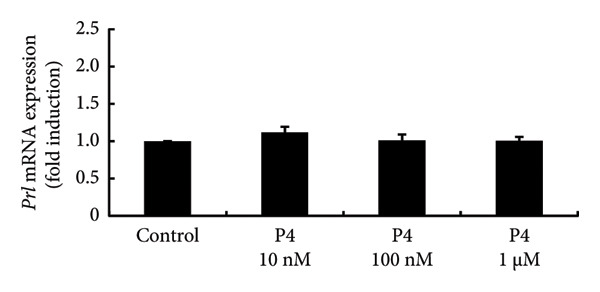
(e)

**Figure:**
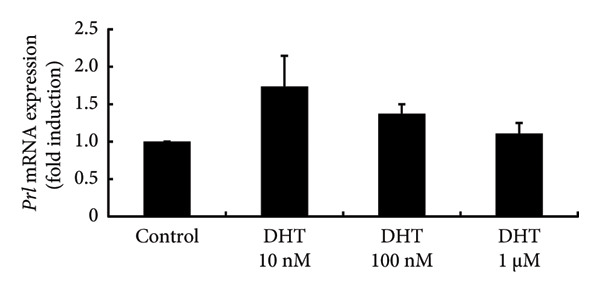
(f)

### 3.5. Effects of OVX on TRH and Dopamine Expression in the Rat Hypothalamus and Their Receptor Expression in the Anterior Pituitary Gland

Hypothalamic TRH is a known secretagogue of PRL, while dopamine is a PRL‐inhibiting hypothalamic factor. We examined the mRNA expression of *Trh* and *Th*, which is a rate‐limiting step of dopamine synthesis within hypothalamic lesions. *Trh* and *Th* expressions in hypothalamic lesions were unchanged by OVX. Their mRNA levels in OVX rat hypothalamus were not altered by E2 supplementation (Figures 5(a) and 5(b)). Following OVX, *Trhr* expression within the anterior pituitary was unchanged compared to control rats. E2 supplementation after OVX had no effect on *Trhr* expression within the anterior pituitary (Figure 5(c)). Similarly, *D2r* expression in the anterior pituitary was not altered by OVX or subsequent E2 supplementation (Figure 5(d)).

Figure 5Effects of OVX and E2 replacement on *Trh* expression in the hypothalamus. Female rats aged 6 weeks underwent OVX, or received subcutaneous implantation of a 0.25 mg E2 pellet following OVX (OVX + E2). Sham‐operated (ovarian‐preserved) rat group was used as the control group. Seven days later, the rats were euthanized, and the anterior (a) and posterior (b) regions of the hypothalamus as well as the anterior pituitary gland (c, d) were excised. Total mRNA was extracted from the tissues and reverse‐transcribed. *Trh* (a) and *Th* (b) mRNA levels in the hypothalamus and *Trhr* (c) and *D2r* (d) mRNA levels in the pituitary gland were measured by quantitative RT‐PCR. Samples from each experimental group were processed in duplicate and normalized based on mRNA levels of the housekeeping gene *Gapdh*. Results are expressed as fold induction relative to the control group and are presented as mean SEM.

**Figure:**
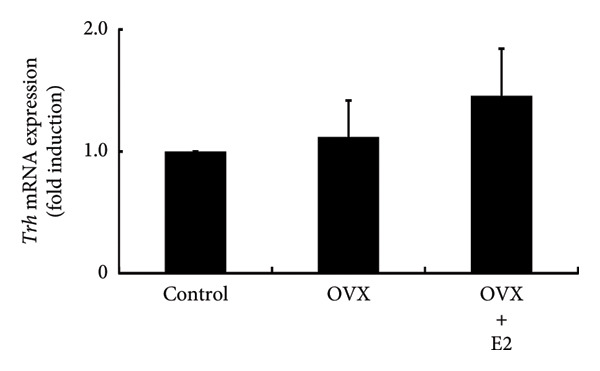
(a)

**Figure:**
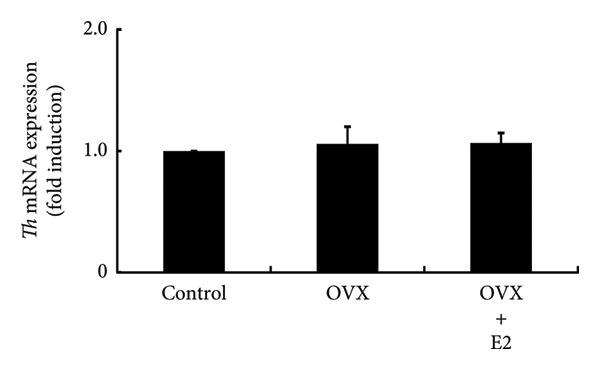
(b)

**Figure:**
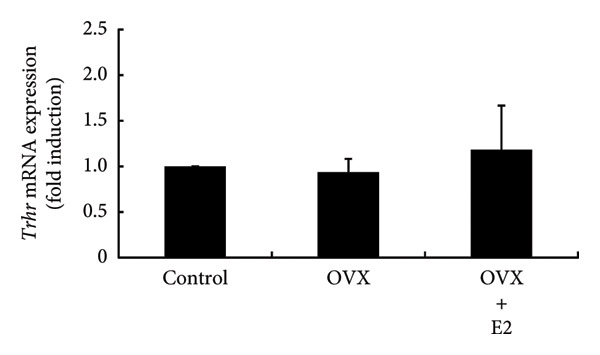
(c)

**Figure:**
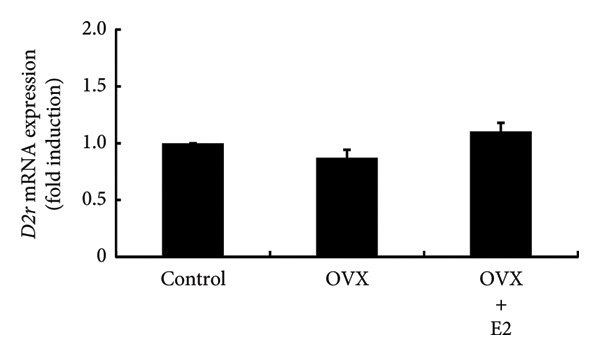
(d)

### 3.6. Effects of OVX on Fst Expression Within the Pituitary Gland

**Figure 6:**
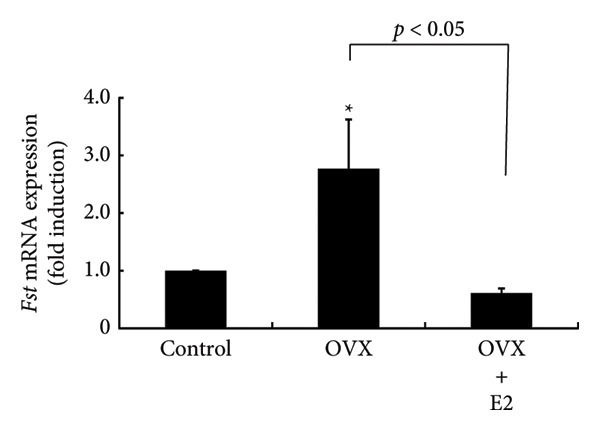
Effects of OVX and E2 replacement on *Fst* mRNA expression in the anterior pituitary gland. Female rats aged 6 weeks underwent OVX, or received subcutaneous implantation of a 0.25 mg E2 pellet following OVX (OVX + E2). Rats that underwent sham surgery (ovaries retained) served as the control group. Seven days later, the rats were euthanized and the anterior pituitary was removed. Total mRNA was extracted from the tissues and reverse‐transcribed. *Fst* mRNA levels were measured by quantitative RT‐PCR. Samples from each experimental group were analyzed in duplicate and normalized against the mRNA level of the endogenous gene *Gapdh*. The results are expressed as fold induction over control and presented as the mean ± SEM. Statistical significance was determined at ^∗^ *p* < 0.05 compared to control. The difference in *Fst* mRNA expression between OVX and OVX + E2 rats was significant (*p* < 0.05).

*Fst* mRNA expression was significantly increased by 2.77 ± 0.85‐fold after OVX (*p* < 0.05) within the pituitary gland. E2 supplementation after OVX completely prevented this increase in *Fst* expression (*p* < 0.05) (Figure 6).

### 3.7. Effects of FST on PRL Expression

Lastly, we examined the effect of FST on PRL synthesis using GH3 cells. TRH significantly increased *Prl* promoter activity by 4.26 ± 0.55‐fold (*p* < 0.01). FST itself did not significantly modulate *Prl* promoter activity and had no effect on the TRH‐induced increase in *Prl* promoter activity (Figure 7(a)). Although *Prl* mRNA expression was increased by TRH (*p* < 0.01), FST did not modulate its expression and had no effect on the TRH‐induced increase in *Prl* mRNA expression (Figure 7(b)).

Figure 7Effects of FST on *Prl* promoter activity and mRNA expression in GH3 cells. (a) GH3 cells were transfected with 2.0 μg *Prl*‐Luc and 0.1 μg pRL‐TK. At 48 h after transfection, the cells were treated with the 100 nM TRH, 10 mg/mL of FST, and both TRH and FST for 6 h. A luciferase assay was performed to evaluate the *Prl* promoter activity. This activity was normalized by *Renilla* luciferase activity and expressed as a fold increase in activation relative to the unstimulated control group. (b) GH3 cells were stimulated with 100 nM TRH, 10 mg/mL FSH, and both TRH and FSH for 24 h. Following stimulation, mRNA was extracted and reverse‐transcribed, and *Prl* mRNA levels were measured by quantitative RT‐PCR. Samples from each experimental group were analyzed in duplicate and normalized against the mRNA level of the housekeeping gene *Gapdh*. The results are expressed as fold induction over unstimulated cells. Data are expressed as the mean ± SEM (three independent experiments were performed using triplicate samples). Statistical significance was determined at ^∗∗^ *p* < 0.01 compared to control.

**Figure:**
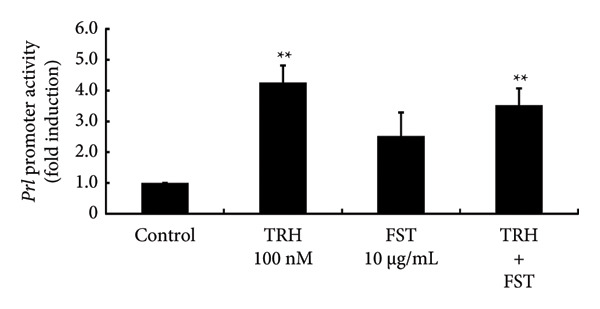
(a)

**Figure:**
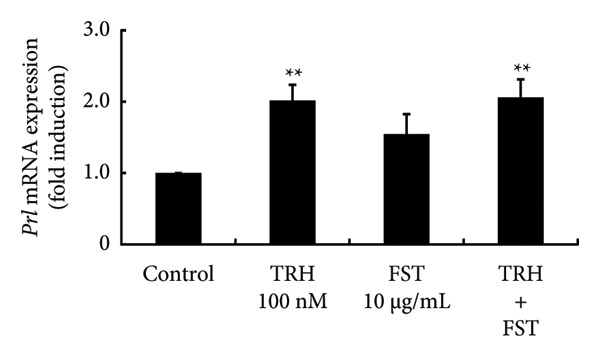
(b)

## 4. Discussion

The HPG axis maintains the female reproductive function through negative feedback mechanism. That is, an excess of E2 reduces pituitary gonadotropin release by inhibiting GnRH release from the hypothalamus and which is believed to be mediated by kisspeptin neurons [14]. It has long been recognized that the hypersecretion of PRL suppresses GnRH release [32]. In addition, kisspeptin neurons express sufficient levels of the PRL receptor, although it is only minimally expressed on the surface of GnRH neurons [33, 34]. These observations indicate that circulating PRL and the HPG axis are strongly related.

Because OVX profoundly affects the HPG axis by completely depleting circulating E2, we examined the effect of OVX on pituitary PRL expression to elucidate the relationship between PRL and the HPG axis. To our surprise, we found that OVX strongly suppressed PRL expression in the pituitary gland. On the other hand, E2 supplementation after OVX completely prevented this decrease in PRL expression. This phenomenon suggests that, similar to gonadotropins, PRL is under the feedback control of E2. Rather than a negative feedback mechanism by E2, PRL seems to be controlled positively by the presence of E2. Among the three sex steroids, E2, P4, and DHT, only E2 prevented the decrease in PRL expression and secretion after OVX. P4 and DHT supplementation after OVX did not prevent the decrease in PRL; in other words, they did not increase PRL expression in the pituitary gland. If the decrease of PRL induced by OVX is controlled by a hypothalamic factor, its upstream factor seemed to sense only E2. In this connection, kisspeptin neurons in the arcuate nucleus, which is believed to be a sensor of sex steroids, express the receptors for E2, P4, and DHT [15, 35].

In the experiments using ovary‐intact rats, E2 administration increased PRL expression. This observation was consistent with the results in OVX rats in which E2 supplementation increased PRL expression after OVX. The administration of P4 and DHT failed to modulate PRL expression, indicating that neither increases PRL levels, even when basal E2 levels are maintained. These results were also consistent with the finding that neither P4 nor DHT prevented the OVX‐induced decrease in PRL expression. Animal experiments indicated that E2 was the only sex steroid that could increase PRL expression. Experiments using PRL‐producing GH3 cells also clearly showed that only E2 promotes PRL production. Although E2 failed to stimulate PRL promoter activity, it was found to stimulate gene expression of PRL. This discrepancy might be due to the absence of the E2 response element in the PRL promoter region used in our experiment. E2 administration to ovary‐intact rats increased PRL, whereas P4 and DHT had no effect. In addition, P4 and DHT did not affect PRL production in GH3 cells, and thus P4 and DHT might not be steroid hormones that have a significant impact on PRL production in vivo. From the observation that OVX abruptly reduced PRL levels and that E2 stimulated PRL expression, E2 might play a role in maintaining PRL expression and secretion. Normal pituitary lactotrophs can be enlarged during pregnancy [36], and E2 administration causes an increase in PRL synthesis [37]. These previous studies support our hypothesis for the role of E2 in PRL expression. PRL exerts a negative feedback effect on its own secretion by indirectly stimulating hypothalamic dopamine secretion [3]. In addition, it also exerts a direct negative feedback effect on lactotrophs in an autocrine or paracrine manner [38, 39]. This is evidenced by the development of hyperprolactinemia and lactotroph adenoma (prolactinoma) in D2R knockout mice or PRL receptor knockout mice [39, 40]. On the other hand, TRH is an endogenous factor that has an impact on PRL secretion [41]. Therefore, we examined the effect of OVX on TRH and dopamine expression in the hypothalamus. *Th* gene was targeted to determine the effect on dopamine because TH is a rate‐limiting step of dopamine synthesis in the hypothalamus [42]. The lack of an effect of OVX on the hypothalamic expression of *Trh* and *Th* suggested that the alteration in hypothalamic TRH or dopamine expression was not responsible for the decrease in pituitary PRL expression after OVX. Furthermore, because the expression of TRH and dopamine receptors was not altered in the pituitary after OVX, it is plausible that these two PRL‐regulating hypothalamic factors and their receptor expression within the pituitary gland were not involved in the change in PRL levels after OVX. It is considered that the decrease in PRL is simply due to the reduction of E2, which maintains the basal secretion of PRL.

The depletion of E2 by OVX is known to induce a drastic increase in pituitary gonadotropins by a negative feedback mechanism, although E2 does increase the levels of some gonadotropin subunits, especially common glycoprotein α and LHβ subunits [21, 43]. This feedback mechanism is believed to be mediated by kisspeptin neurons, called KNDy neurons, which are located in the arcuate nucleus of the hypothalamus [14, 15]. Although hypothalamic GnRH is the principal regulator of pituitary gonadotropin synthesis and secretion, the activin/inhibin/FST system also works locally within the pituitary gland and regulates FSH synthesis [44, 45]. We have previously reported that FST expression within the pituitary gland is increased by OVX and this increase can be repressed by E2 supplementation after OVX [43]. In the present study, we again confirmed this phenomenon and examined the effects of FST on PRL synthesis. TRH was applied as a secretagogue for PRL. FST itself did not modulate *Prl* promoter activity or the basal expression of *Prl* mRNA. These observations suggest that even if locally expressed FST is increased by OVX, it does not seem to have a significant impact on the expression of pituitary PRL. In other words, only the decrease in E2 is likely to cause the reduction in PRL due to OVX and this phenomenon is not mediated by hypothalamic neurons such as kisspeptin, TRH, and dopamine neurons.

Based on the findings of this study, we speculated that reduction of E2 by OVX is only one cause of the reduction of PRL in female rats, given that the levels of PRL‐inhibiting or PRL‐stimulating factors as well as their receptors in the pituitary gland were unchanged after OVX. Observations that E2 supplementation prevented a decrease in E2 after OVX, that E2 was the sole sex steroid that enhanced PRL expression in ovary‐intact female rats, and that only E2 increased PRL gene expression in PRL‐producing GH3 cells support this hypothesis. This study has several limitations. The effect of OVX on hypothalamic factors has not been analyzed at the protein level, and gene expression was analyzed using only one part of the hypothalamus. Therefore, it is necessary to examine the expression levels of TRH or dopamine in a wider range of areas within the brain after OVX. We speculate that P4 and DHT do not play an important role in PRL production, but it is possible that PRL levels were elevated due to unphysiological high concentrations of E2 stimulation in vivo and in GH3 cells. Thus, it is necessary to examine whether a more physiological concentration of E2 suppresses the decrease in PRL caused by OVX. Furthermore, regarding P4 and DHT, a more detailed examination of the stimulation concentrations and an investigation of sex steroid hormone receptor quantities need to be conducted.

## 5. Conclusion

In this study, we revealed that PRL expression was decreased by OVX and increased by E2 supplementation after OVX in female rats. This phenomenon was not influenced by hypothalamic factors or the expression of their receptors in the pituitary gland. We speculated that due to the reduction in E2 levels by OVX, PRL, whose basal expression is maintained by E2, was decreased.

## Ethics Statement

All applicable international, national, and/or institutional guidelines concerning the care and use of animals were followed.

## Disclosure

All authors listed have reviewed and approved the publication of this paper.

## Conflicts of Interest

The authors declare no conflicts of interest.

## Author Contributions

T.T. and H.K. designed the research; Z.C., S.S.Y., T.T., A.O., and H.O. performed the research and analyzed data; Z.C. and H.K. wrote the paper; S.K. supervised the research.

## Funding

This work was supported in part by a Grant‐in‐Aid for Scientific Research from the Ministry of Education, Culture, Sports, Science and Technology of Japan (23K08798, to H.K.).

## Data Availability

The datasets used and/or analyzed during this study are available from the corresponding authors upon reasonable request.
